# Engagement in physical education classes and health among young people: does sports practice matter? A cross-sectional study

**DOI:** 10.1590/1516-3180.2017.0111260617

**Published:** 2017-11-17

**Authors:** Diogo Henrique Constantino Coledam, Philippe Fanelli Ferraiol

**Affiliations:** I MSc, PhD. Physical Educator and Professor, Instituto Federal de Educação, Ciência e Tecnologia de São Paulo (IFSP), Boituva (SP), Brazil.; II MSc. Physical Educator and Doctoral Student, Universidade Estadual de Londrina (UEL), Londrina (PR), Brazil.

**Keywords:** Physical fitness, Exercise, Obesity, Students

## Abstract

**CONTEXT AND OBJECTIVE:**

Physical education classes aim to promote health but it is unknown whether benefits occur independently of sports practice. The purpose of this study was to examine associations between engagement in physical education classes and physical fitness and obesity according to sports practice among Brazilian students.

**DESIGN AND SETTING:**

Cross-sectional school-based study involving 737 students aged 10-17 years in southern Brazil.

**METHODS:**

Engagement in physical education classes and sports practice were analyzed using a self-report questionnaire. The health indicators analyzed were cardiorespiratory fitness, muscle strength, obesity and combinations thereof. The covariates were sex, age, socioeconomic status, physical activity and sedentary behavior. Prevalence ratios (PR) adjusted for confounding variables were estimated using Poisson regression. Analyses were stratified according to sports practice.

**RESULTS:**

Engagement in physical education classes was associated with achievement of health-related criteria for cardiorespiratory fitness (PR = 1.52), muscle strength (PR = 1.55), obesity + cardiorespiratory fitness (PR = 1.51), obesity + muscle strength (PR = 1.70), cardiorespiratory fitness + muscle strength (PR = 2.60) and the three outcomes combined (PR = 2.43), only among non-sports practitioners, all P < 0.05. Engagement in physical education classes was not associated with obesity (PR = 1.00, P > 0.05). No associations were found for sports practitioners (P > 0.05).

**CONCLUSION:**

Engagement in physical education classes was associated with health among non-sports practitioners. However, to protect students from obesity and promote additional health benefits for sports practitioners, the conventional physical education program offered to the sample studied should be reformulated.

## INTRODUCTION

Physical activity is an important health-related behavior in different age groups. It is associated with mental, cardiovascular and bone health, lower adiposity, greater physical fitness, greater motor skills development and better quality of life among children and adolescents.^1^ Approximately 70% of Brazilian adolescents are inactive and therefore promotion of physical activity in this age group is necessary.^2^ In this context, since schools have a responsibility for promoting physical activity, the subject of physical education takes on an important role in relation to public health among young people,^3^ with the aims of increasing the amount of moderate to vigorous physical activity and decreasing students’ daily sedentary behavior.^4^ In addition, physical education classes have the goal of providing students with knowledge, skills and confidence so that they can be active throughout their lifetime, thus preventing the emergence of health problems.^3^^,^^5^

Several observational and experimental studies have been conducted to examine the benefits of physical education classes for schoolchildren’s health. Physical fitness and obesity are widely investigated outcomes because of their contribution to cardiovascular, metabolic, musculoskeletal and mental health among young people.^6^^,^^7^ Experimental studies have demonstrated that intervention programs within physical education classes increase muscle strength^8^^,^^9^ and cardiorespiratory fitness,^8^^,^^9^^,^^10^ and decrease the body mass index^8^ and prevalence of overweight^11^ among children and adolescents. Similarly, an observational study that evaluated 91,236 fifth-grade students in California, United States, found that policies offering physical education classes were associated with better cardiorespiratory fitness.^12^

In analyzing the relationship between physical education programs and health among young people, sports practice is a variable that needs to be considered for two reasons. Firstly, sports practice is associated with habitual physical activity among young people^13^^,^^14^ and consequently increases their cardiorespiratory fitness and muscle strength,^13^^,^^15^^,^^16^ and decreases their overweight and obesity.^17^ Secondly, it has recently been reported that the effects of physical education classes on cardiorespiratory fitness occur only among young people in a poor physical condition,^10^ who are probably not sports practitioners. Despite the information provided, studies conducted so far with the aim of examining the relationship between physical education classes and health, as well as those analyzing the effects of intervention programs within physical education classes, have not considered whether the participants were sports practitioners. This limitation prevents knowledge of whether the benefits of physical education classes on health occur among both young people who practice and those who do not practice sports in their leisure time.

## OBJECTIVE

The aim of the present study was to examine associations of engagement in physical education classes with physical fitness and obesity, according to sports practice, among Brazilian young people.

## METHODS

### Ethics

This study was approved by the Ethics Committee for Research Involving Human Beings of the State University of Londrina (Universidade Estadual de Londrina, UEL), Paraná, Brazil, under protocol 312/2011. A parent or legal guardian provided written informed consent through signing a statement in which the aims of the study, details about the procedures, risks and benefits of the study and contact details of the researcher were described.

### Study sample and design

This was a cross-sectional study that formed part of a larger epidemiological survey entitled “Physical education and health criteria achievement in Brazilian young people”, which was conducted from May to July 2012. The aim of the larger survey was to investigate the association between engagement in physical education classes and health indicators in a representative sample of students in the city of Londrina, Paraná, Brazil.

The study population was composed of students enrolled in state schools in Londrina in 2012. The inclusion criteria were that they needed to:

1. Agree voluntarily to participate in the study;
2. Provide an informed consent statement signed by a legal representative;
3. Be aged between 10 and 17 years;
4. Be enrolled in a state school;
5. Not present any physical or metabolic limitations that would prevent performance of any study procedures; and
6. Undergo all the proposed procedures.

For this study, the sample size was estimated considering a population of 55,475, outcome prevalence of 40%, confidence interval of 95%, design effect of 2, and sample loss of 30%, using the Epi Info 7.0 software. The minimum sample size was estimated as 732 students.

Out of the 965 students invited to participate in this study, 737 met the eligibility criteria and composed the final sample. The students were aged 10 to 17 years and were probabilistically selected through clusters (school and classrooms) that were stratified according to region of the city (north, south, east, west and center), sex and school year. The sampling procedure was performed in two stages. One school from each region of the city was selected randomly and the proportional number of students in the region was assessed using full classrooms (25-30 students).

### Data collection and variables

All procedures were carried out at the school in which the participants were enrolled. The questionnaire was answered and the anthropometric measurements were performed in the classroom. The field tests were carried out in the school’s indoor sports court. All information was collected within a maximum period of three days.

The independent variables of the present study were sports practice and engagement in physical education classes. Sports practice was analyzed by means of the following question: “In leisure time activities, do you practice sports?”, with the following response options: never; rarely; sometimes; frequently; or always. The question was taken from the Questionnaire of Habitual Physical Activity*.*^18^ Participants who answered “frequently” or “always” were considered to be sports practitioners*.*

Engagement in physical education classes was assessed using two self-report questions:

1. In this semester, did you participate in physical education classes?”, with answer options “no”, “yes, but only one class per week” or “yes, I participated in all classes”. This question presented 90% agreement one month later, through direct observation among 40 students selected from the sample of the present study.

2. “Generally, during physical education classes, how active were you, i.e. did you play, run, jump and throw balls intensely?” with the following response options: “I didn’t participate in the classes,” “rarely,” “sometimes,” “often” or “always.” This question was adapted from the PAQ-C questionnaire (Physical Activity Questionnaire for Children).

The translation and cross-cultural adaptation of PAQ-C into the Portuguese language, and its reproducibility and concurrent validity, have been described elsewhere.^19^ We tested the validity of question 2 for assessing the intensity of classes, by using a perceived exertion scale^20^ in eight physical education classes: two classes a week for one month. Students who reported being active during classes presented significantly higher perceived exertion than did those who reported not being active: 4.0 (3.0-5.0) versus 6.5 (4.5-7.5) arbitrary units; P < 0.05. Participants who answered that they had participated in all physical education classes and were “often” or “always” active during classes were considered to be engaged in physical education classes. The independent variables presented high reproducibility (agreements of 80% and 93.4%).

### Outcomes

The outcomes of the study were cardiorespiratory fitness, muscle strength, obesity and combinations of these outcomes. Cardiorespiratory fitness was evaluated by means of the multistage 20-meter shuttle run test.^21^ This is a progressive test and participants are required to run back and forth over a 20-meter distance. The velocity starts at 8.5 km/h and increases by 0.5 km/h each minute until voluntary exhaustion. Upper-limb muscle strength was estimated using the 90° push-up test.^22^ The cutoffs used for cardiorespiratory fitness and muscle strength (health fitness zone) were as proposed through Fitnessgram, according to sex and age.^22^ Nutritional status was assessed through the body mass index (BMI = body mass/height^2^). Measurements of body mass and height were obtained using a digital scale and a portable stadiometer. The cutoff points used to classify obesity were as proposed by the International Obesity Task Force.^23^ The above outcomes were analyzed both separately and in combinations.

### Covariates

The covariates used to adjust the analysis were sex, age, socioeconomic condition, physical activity and sedentary behavior. Socioeconomic condition was estimated using the questionnaire of the Brazilian Association of Polling Companies. The Questionnaire of Habitual Physical Activity was used to assess physical activity.^18^ The following question was used to assess sedentary behavior: “How many hours on average do you watch TV, play video games or use the computer,” with the following response options: < 1 hour per day, 1 hour per day, 2 hours per day, 3 hours per day, 4 hours per day or 5 or more hours per day.

### Physical education curriculum

In the year during which the study was conducted, the school subject of physical education was taught by a physical education teacher and each student had timetabled classes totaling 100 minutes/week. All schools included in the sample had an indoor sports court. In the state schools of the state of Paraná, the physical education curriculum has the objective of teaching body culture, which is based on the cultural forms of human movement historically produced by humanity. This is based on the assumption that the pedagogical practice of physical education within the school context should turn the different forms of body expression activities into topics, consisting of the following: games, sports, gymnastics, rhythmic activities and martial arts. With the aims of increasing knowledge of reality and establishing relationships between everyday social and cultural phenomena, the curriculum also includes the following articulating elements: body, playfulness, health, world of work, technical and tactical elements, leisure time, diversity and media. This curriculum is the same as in other Brazilian states and details have previously been described.^24^

### Statistical analyses

Descriptive statistics were produced, comprising relative frequencies and 95% confidence intervals. The chi-square test was used to analyze the bivariate association between engagement in physical education classes and health indicators. Multivariate analysis was performed using Poisson regression to estimate prevalence ratios (PR) and 95% confidence intervals. The analyses were stratified according to sports practice and independent variables were inserted simultaneously in the final model. Because of the complex sample used and stratifications of the analysis according to sports practice, the multivariate analysis was conducted considering the strata, primary sample units and sample weight, using the “survey” (svy) command of STATA 11.0. In all cases, results were considered significant when P < 0.05.

## RESULTS

The sample loss from the present study was 23.6%. This loss arose because some of the students did not perform all the study procedures. However, this did not affect the representativeness of the sample, given that missing values had been anticipated in the sample size estimates, and because the losses did not change the proportions among the participants according to sex, age, socioeconomic status or region of the city. Moreover, the losses did not prevent the study from attaining the minimum sample size required to conduct the analysis.

Out of the 737 students, 35% reported practicing sports. Higher proportions of the sports practitioners were males, were aged between 10 and 12 years, were engaged in physical education classes and achieved the health criteria for cardiorespiratory fitness and muscle strength (P < 0.05). No differences between the proportions of sports practitioners and non-practitioners regarding socioeconomic status or obesity were found (P > 0.05) (Table 1).

**Table 1.**
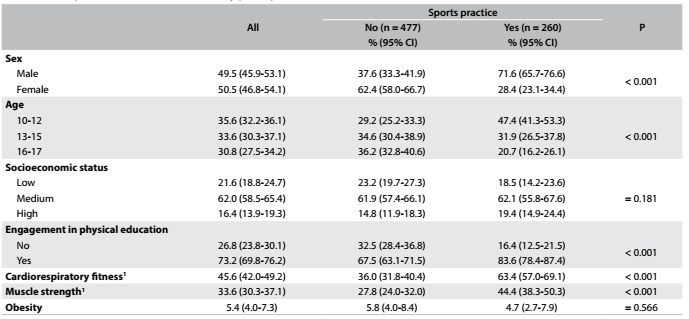
Descriptive characteristics of the study participants (n = 737)

^1^Achievement of health status according to the criteria proposed through Fitnessgram; 95% CI = 95% confidence interval of prevalence; P refers to the chi-square test.

The bivariate analyses are presented in Table 2. Among the young people who did not practice sports, positive associations were found between engagement in physical education classes and health criterion achievement regarding cardiorespiratory fitness and muscle strength (P < 0.05). No association was found regarding obesity (P > 0.05). In analyzing combined outcomes, positive associations were found between engagement in physical education classes and the following variables: obesity + cardiorespiratory fitness, obesity + muscle strength, cardiorespiratory fitness + muscle strength and all outcomes combined (P < 0.05). No association was found between engagement in physical education classes and achievement of health criteria for any of the outcomes analyzed, for the participants who were sports practitioners (P > 0.05).

**Table 2.**
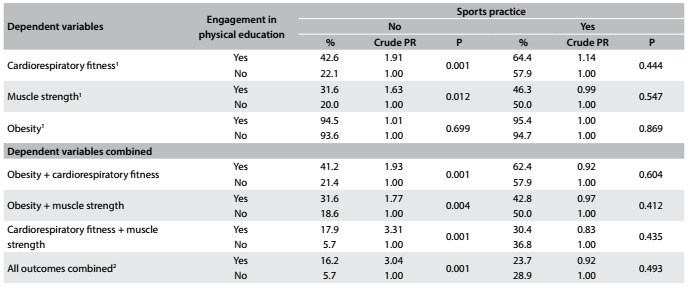
Bivariate association analysis between engagement in physical education classes and achievement of health criteria among students

PR = prevalence ratio; % = relative frequency; P = P for chi-square test; ^1^Achievement of health status according to the criteria proposed through FitnessGram; ^2^Health criteria combined (cardiorespiratory fitness, muscle strength and obesity).

The results described in the bivariate analysis were maintained after adjustment for the confounding variables (Table 3). Young people who reported not practicing sports but being engaged in physical education classes were more likely to achieve the health criteria for cardiorespiratory fitness (PR = 1.52), muscle strength (PR = 1.55), obesity + cardiorespiratory fitness (PR = 1.51), obesity + muscle strength (PR = 1.70), cardiorespiratory fitness + muscle strength (PR = 2.60) and all outcomes combined (PR = 2.43), all with P < 0.05. Being engaged in physical education classes was not associated with obesity for those who were not sports practitioners or with any of the outcomes for those who were sports practitioners (P > 0.05).

**Table 3.**
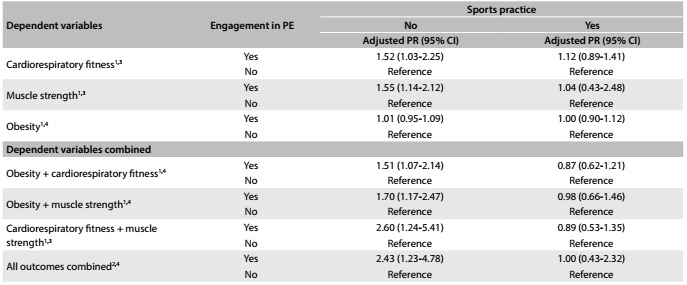
Multivariate analysis on the association between engagement in physical education (PE) classes and achievement of health criteria among students

PR = prevalence ratio; 95% CI = 95% confidence interval; ^1^Achievement of health status according to the criteria proposed through FitnessGram; ^2^Health criteria combined (cardiorespiratory fitness, muscle strength and obesity); ^3^Adjusted for sex, age, socioeconomic status, obesity, physical activity and sedentary behavior; ^4^Adjusted for sex, age, socioeconomic status, physical activity and sedentary behavior.

## DISCUSSION

The aim of this study was to analyze the association between engagement in physical education classes and some health indicators, according to sports practice among young Brazilians. The novelty of this study was that engagement in physical education classes was associated with cardiorespiratory fitness, muscle strength and combined health outcomes, only among students who did not practice sports. In contrast, engagement in physical education classes was not associated with obesity, independent of sports practice.

Although the present study had a cross-sectional design, the results found regarding participants who were not sports practitioners corroborate previous experimental studies that demonstrated increases in cardiorespiratory fitness^8^^,^^9^^,^^10^ and muscle strength^8^^,^^9^ after implementation of intervention programs within physical education classes. Likewise, they corroborate an observational study that was carried out on a representative sample in the American state of California. The results from that study demonstrated that adoption of public policies to promote physical education classes was associated with higher cardiorespiratory fitness among schoolchildren.^12^ Despite the information available regarding the relationship between physical education programs and health, none of the studies listed above considered students’ sports practice, which limits comparison of the results.

Differently from previous studies, it was sought in the present study to investigate whether engagement in physical education classes was associated with health indicators, among both young people who practiced sports and those who did not. There was no benefit in engaging in physical education classes for the students who were sports practitioners, in relation to any of the variables analyzed. This can probably be explained in terms of the adaptations resulting from the intensity of sports practice. Although sports practice during leisure time was analyzed in the present study, this type of activity is usually performed at high intensities and results in positive cardiovascular and muscle adaptations.^25^ Associations between sports practice and cardiorespiratory fitness,^13^^,^^16^ muscle strength^15^ and protection against overweight and obesity have been described.^17^ Because young people who practice sports are more active,^13^^,^^14^ they are probably protected from the outcomes analyzed in the present study. Hence, engagement in physical education classes would not present any additional benefit. One previous result that reinforces this statement was the finding that a physical education program only increased cardiorespiratory fitness among young people who were in a poor physical condition,^10^ who probably were not practicing sports or physical exercise. The results from the present study also indicate that there is a need to estimate sports practice when analyzing the effects of both intervention programs and conventional physical education on the health of young people, in order to better understand the results. However, this methodological procedure is not commonly performed.

In the present study, the outcomes were analyzed separately and in combinations. Analysis on combined outcomes is necessary for two reasons. Firstly, in combining low physical fitness with indicators of high adiposity, there is an increase in cardiovascular risk in comparison with the separate outcomes.^26^^,^^27^ Secondly, cardiorespiratory fitness, muscle strength and low adiposity are independently associated with cardiometabolic risk^28^^,^^29^^,^^30^ and thus, it is desirable that young people should fulfill all of these health criteria. Thus, the results showed that engagement in physical education classes was associated with higher probability of achievement of combined health criteria among participants who were not sports practitioners.

It is worth noting that it was difficult to compare the results found with those previously reported, since it has been unusual for studies investigating relationships between physical education classes and health to use combined outcomes. From a public health point of view, this is an important result, given that sports make a large contribution towards total physical activity.^13^^,^^14^ Students who do not practice sports may perform less daily physical activity and be exposed to the risks of future non-communicable diseases.^2^

Regarding the results relating to physical fitness and combined outcomes, it had been expected that engagement in physical education classes would be associated with obesity among students who were not sports practitioners, but this did not occur. This result corroborates a previous study that demonstrated that school physical education, as typically offered, does not reduce or prevent obesity.^31^ Although it has the potential to assist in controlling obesity, there is a need to implement stricter policies to promote physical education.^32^ Another point that should be highlighted is that strategies for promoting physical activity in isolation do not demonstrate efficacy in reducing obesity. Obesity-related outcomes were found to be improved in intervention programs with two or three components (i.e. physical activity plus nutrition and physical activity plus both education and nutrition),^33^^,^^34^ which did not occur in conventional physical education programs as analyzed in the present study.

The present study has limitations that need to be considered in interpreting the results. Although previous studies provided the theoretical basis for demonstrating the effects of physical education classes on students’ health, the design used here was cross-sectional, which prevented inferences regarding causality among the associations identified. The main limitation was that engagement in physical education classes was estimated through a self-report questionnaire, which prevented accurate measurement of the intensity of classes, in comparison with objective measurements. However, this limitation was attenuated, given that the instrument used was valid for detecting students who reported higher perceived exertion during classes. Regarding sports practice, although the instrument used showed correlations with the amount of daily physical activity,^35^ it presented the limitation of not estimating which types of sports were practiced by these young people, or the volume and intensity of the activities. Despite these limitations, the present study had a representative sample and analyzed a conventional physical education program with outcomes relating to public health, using multivariate analysis. This enables generalization of the results to populations with similar characteristics and similar physical education programs.

Physical education plays an important role in health promotion among young people who do not practice sports, because of the protection that it provides against the risk of low physical fitness. However, to provide protection against obesity and obtain additional benefits regarding the health of young sports practitioners, conventional Brazilian physical education programs require improvement. Future studies aiming towards examining the relationship between physical education programs and health should consider sports practice, in order to better understand the benefits among young people.

## CONCLUSION

The benefits of engagement in physical education classes regarding cardiorespiratory fitness and muscle strength were only seen among students who did not practice sports. On the other hand, no association was observed regarding obesity. Benefits were also observed when the variables of cardiorespiratory fitness, muscle strength and obesity were combined for analysis.
